# (Acetyl­acetonato)(dicyanamido)(1,10-phenanthroline)copper(II) dihydrate

**DOI:** 10.1107/S1600536810030163

**Published:** 2010-08-04

**Authors:** Halimeh Janani, Ali Reza Rezvani, Faramarz Rostami-Charati, Mohamed Makha, Brian W. Skelton

**Affiliations:** a Department of Chemistry, University of Sistan and Baluchestan, PO Box 98135-674, Zahedan, Iran; b Faculty of Science, Gonbad Higher Education Center, PO Box 163, Gonbad, Iran; c Chemistry, School of Biomedical, Biomolecular & Chemical Sciences, The University of Western Australia, 35 Stirling Highway, Crawley, Perth, Western Australia 6009, Australia

## Abstract

In the title compound, [Cu(C_5_H_7_O_2_)(C_2_N_3_)(C_12_H_8_N_2_)]·2H_2_O, the Cu^II^ atom is five-coordinated in a square-pyramidal geometry with two acetyl­acetonate O and two phenanthroline N atoms forming the base. The apical position is occupied by the central N atom of the dicyanamide ligand. The dicyanamide N atoms are each involved in hydrogen bonds to water mol­ecules. There are also hydrogen bonds between both the water mol­ecules and their centrosymmetric pairs, creating a hydrogen-bonded chain along the *b*-axis direction.

## Related literature

Dicyanamide (dca) has been shown to be a versatile ligand and may coordinate to metal ions as a terminal ligand through a nitrile or amide nitro­gen. It also acts as a bridging ligand. Until now, as many as eight structurally characterized coordination modes of dicyanamide had been reported in the literature, see: Chattopadhyay *et al.* (2008 ▶); Liu *et al.* (2005 ▶); Miller & Manson (2001 ▶); Xu *et al.* (2003 ▶).
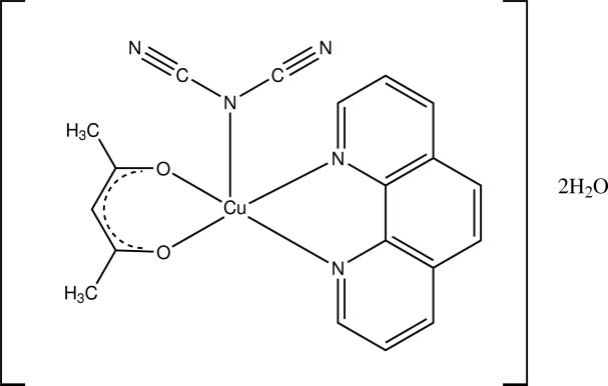


## Experimental

### 

#### Crystal data

[Cu(C_5_H_7_O_2_)(C_2_N_3_)(C_12_H_8_N_2_)]·2H_2_O
*M* *_r_* = 444.93
Triclinic, 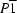

*a* = 8.2825 (8) Å
*b* = 9.9853 (7) Å
*c* = 12.1109 (7) Å
α = 76.388 (5)°
β = 79.236 (7)°
γ = 83.554 (7)°
*V* = 953.90 (13) Å^3^
*Z* = 2
Mo *K*α radiation
μ = 1.18 mm^−1^
*T* = 100 K
0.44 × 0.38 × 0.15 mm

#### Data collection

Oxford Diffraction Gemini diffractometer
Absorption correction: analytical (*CrysAlis RED*; Oxford Diffraction, 2009 ▶) *T*_min_ = 0.667, *T*_max_ = 0.847
10664 measured reflections
6254 independent reflections
4672 reflections with *I* > 2σ(*I*)
*R*_int_ = 0.028

#### Refinement

*R*[*F* ^2^ > 2σ(*F* ^2^)] = 0.034
*wR*(*F* ^2^) = 0.082
*S* = 0.97
6254 reflections
280 parameters
6 restraints
H atoms treated by a mixture of independent and constrained refinement
Δρ_max_ = 0.52 e Å^−3^
Δρ_min_ = −0.42 e Å^−3^

### 

Data collection: *CrysAlis CCD* (Oxford Diffraction 2009 ▶); cell refinement: *CrysAlis RED* (Oxford Diffraction, 2009 ▶); data reduction: *CrysAlis RED*; program(s) used to solve structure: *SIR92* (Altomare *et al.*, 1994 ▶); program(s) used to refine structure: *SHELXL97* (Sheldrick, 2008 ▶); molecular graphics: *ORTEPII* (Johnson, 1976 ▶); software used to prepare material for publication: *publCIF* (Westrip, 2010 ▶).

## Supplementary Material

Crystal structure: contains datablocks I, global. DOI: 10.1107/S1600536810030163/om2347sup1.cif

Structure factors: contains datablocks I. DOI: 10.1107/S1600536810030163/om2347Isup2.hkl

Additional supplementary materials:  crystallographic information; 3D view; checkCIF report

## Figures and Tables

**Table:** 

Cu1—O1	1.9061 (11)
Cu1—O2	1.9072 (11)
Cu1—N1	2.0100 (14)
Cu1—N2	2.0136 (13)
Cu1—N3	2.3920 (15)

**Table:** 

O1—Cu1—O2	95.58 (5)
O1—Cu1—N1	171.80 (5)
O2—Cu1—N1	90.01 (5)
O1—Cu1—N2	91.52 (5)
O2—Cu1—N2	168.73 (5)
N1—Cu1—N2	82.08 (5)
O1—Cu1—N3	89.74 (5)
O2—Cu1—N3	94.16 (5)
N1—Cu1—N3	95.85 (5)
N2—Cu1—N3	94.62 (5)

**Table 2:** Hydrogen-bond geometry (Å, °)

*D*—H⋯*A*	*D*—H	H⋯*A*	*D*⋯*A*	*D*—H⋯*A*
O2*W*—H2*B*⋯N5	0.81 (2)	2.08 (2)	2.879 (2)	173 (2)
O2*W*—H2*A*⋯O1*W*^i^	0.80 (2)	1.98 (2)	2.761 (2)	167 (2)
O1*W*—H1*A*⋯N4	0.81 (2)	2.10 (2)	2.910 (2)	172 (3)
O1*W*—H1*B*⋯O2*W*^ii^	0.78 (2)	2.00 (2)	2.742 (2)	160 (2)

Symmetry codes: (i) 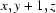; (ii) 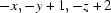.

**Fig. 1.**
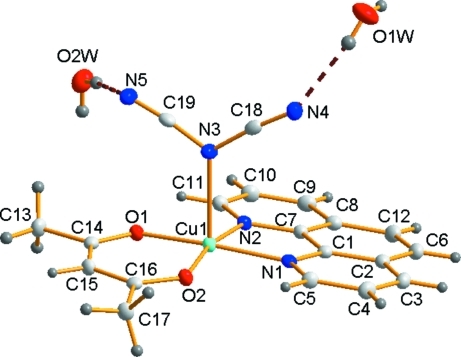
The Molecular structure projected oblique the basal coordination plane. Displacement ellipsoids of non-H atoms are drawn at the 50% probability level. H atoms are drawn as spheres with arbitrary radii.

**Fig. 2.**
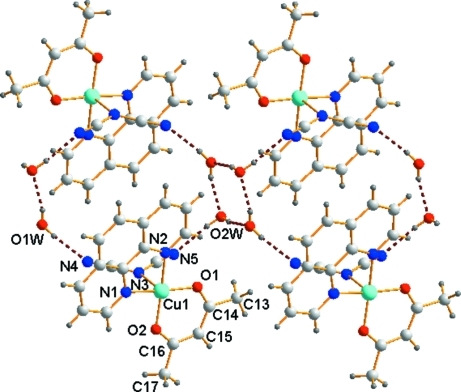
The hydrogen-bonded polymer.

## supplementary crystallographic information

### Comment

Metal dicyanamide (dca) compounds are of great interest due to the variety of observed topologies, this being related to the versatility of dca as a ligand, and its potential application in functional materials. In the present work, we describe the synthesis and crystal structure of a new Cu^II^ complex using the diimine ligand (phen), a bidentate ligand with two oxygen donor atoms (acac) and the anionic co-ligand dicyanamide (dca) (Fig. 1). To date, a number of higher - dimensional coordination networks of different transition metals have been reported with dca as a bridging ligand, but there are few compounds with dca acting as a monodentate ligand through the amide nitrogen. To the best of our knowledge, this complex is one of the few cases where dca is acting as a terminal ligand through the amide nitrogen. The molecule of the title compound is shown in Fig. 1 with selected bond lengths and angles listed in Table 1. In this molecule the coordination is square pyramidal with the two acac O and two phen N atoms forming the base. The apical position is occupied by the N of the dicyanamido ligand with the Cu—N3 distance (Cu1—N3 2.3920 (15) Å) being much greater than those in the basal plane Cu1—O1, 1.906 (1), 1.907 (1) Å and Cu1—N1, 2.010 (1), 2.014 (1) Å. The dicyanamide N atoms, N4, N5 are each involved in hydrogen bonds to water molecules. There are also hydrogen bonds between both the water molecules and their centrosymmetric pairs creating a one dimensional hydrogen bonded polymer in the *b* direction (see Fig. 2). Geometrical details are listed in Table 2.

### Experimental

Acetylacetone (0.103 ml, 1 mmol) was added to a 20 ml methanolic solution of CuCl_2_.2H_2_O (170 mg, 1 mmol). After 30 min of stirring, a solution of phen (198 mg, 1 mmol) in 10 ml methanol was added dropwise to this solution. A solution of 1 mmol of sodium dicyanamide (89 mg) dissolved in 5 ml water was then added slowly with stirring. After 10 h of stirring at room temperature, the resulting solution was filtered to remove any undissolved materials. A dark blue crystalline product separated after 2 weeks.

### Refinement

All H atoms were positioned geometrically and refined using a riding model with C—H = 0.95–0.98 Å and with *U*_iso_(H) = 1.2 times *U*_eq_(C) for CH and *U*_iso_(H) = 1.5 times *U*_eq_(C) for those on terminal C atoms. Anisotropic displacement parameters were employed throughout for the non-hydrogen atoms. Hydrogen atoms on water molecules were located in the difference Fourier map and refined with O-H bond lengths restrained to ideal values.

### Crystal data

**Table:**

[Cu(C_5_H_7_O_2_)(C_2_N_3_)(C_12_H_8_N_2_)]·2H_2_O	*Z* = 2
*M**_r_* = 444.93	*F*(000) = 458
Triclinic, *P*1	*D*_x_ = 1.549 Mg m^−^^3^
Hall symbol: -p 1	Mo *K*α radiation, λ = 0.71073 Å
*a* = 8.2825 (8) Å	Cell parameters from 5220 reflections
*b* = 9.9853 (7) Å	θ = 3.5–32.5°
*c* = 12.1109 (7) Å	µ = 1.18 mm^−^^1^
α = 76.388 (5)°	*T* = 100 K
β = 79.236 (7)°	Slab, blue
γ = 83.554 (7)°	0.44 × 0.38 × 0.15 mm
*V* = 953.90 (13) Å^3^	

### Data collection

**Table:**

Oxford Diffraction Gemini diffractometer	6254 independent reflections
graphite	4672 reflections with *I* > 2σ(*I*)
Detector resolution: 10.4738 pixels mm^-1^	*R*_int_ = 0.028
ω scans	θ_max_ = 32.6°, θ_min_ = 3.5°
Absorption correction: analytical (*CrysAlis RED*; Oxford Diffraction, 2009)	*h* = −12→10
*T*_min_ = 0.667, *T*_max_ = 0.847	*k* = −15→14
10664 measured reflections	*l* = −17→17

### Refinement

**Table:**

Refinement on *F*^2^	Primary atom site location: structure-invariant direct methods
Least-squares matrix: full	Secondary atom site location: difference Fourier map
*R*[*F*^2^ > 2σ(*F*^2^)] = 0.034	Hydrogen site location: inferred from neighbouring sites
*wR*(*F*^2^) = 0.082	H atoms treated by a mixture of independent and constrained refinement
*S* = 0.97	*w* = 1/[σ^2^(*F*_o_^2^) + (0.0386*P*)^2^] where *P* = (*F*_o_^2^ + 2*F*_c_^2^)/3
6254 reflections	(Δ/σ)_max_ = 0.002
280 parameters	Δρ_max_ = 0.52 e Å^−^^3^
6 restraints	Δρ_min_ = −0.42 e Å^−^^3^

### Special details

**Table:**

Geometry. All s.u.'s (except the s.u. in the dihedral angle between two l.s. planes) are estimated using the full covariance matrix. The cell s.u.'s are taken into account individually in the estimation of s.u.'s in distances, angles and torsion angles; correlations between s.u.'s in cell parameters are only used when they are defined by crystal symmetry. An approximate (isotropic) treatment of cell s.u.'s is used for estimating s.u.'s involving l.s. planes.
Refinement. Refinement of *F*^2^ against ALL reflections. The weighted *R*-factor *wR* and goodness of fit *S* are based on *F*^2^, conventional *R*-factors *R* are based on *F*, with *F* set to zero for negative *F*^2^. The threshold expression of *F*^2^ > σ(*F*^2^) is used only for calculating *R*-factors(gt) *etc*. and is not relevant to the choice of reflections for refinement. *R*-factors based on *F*^2^ are statistically about twice as large as those based on *F*, and *R*- factors based on all data will be even larger.The water molecule hydrogen geometries were restrained to ideal values.

### Fractional atomic coordinates and isotropic or equivalent isotropic displacement parameters (Å^2^)

**Table:**

	*x*	*y*	*z*	*U*_iso_*/*U*_eq_	
Cu1	0.44471 (3)	0.77615 (2)	0.641559 (16)	0.01300 (6)	
O1	0.30198 (14)	0.93968 (11)	0.64219 (9)	0.0147 (2)	
O2	0.46649 (15)	0.78689 (11)	0.48019 (9)	0.0151 (2)	
N1	0.61346 (17)	0.61447 (13)	0.65445 (11)	0.0131 (3)	
N2	0.47043 (17)	0.76076 (13)	0.80625 (11)	0.0136 (3)	
N3	0.20994 (18)	0.64152 (14)	0.69007 (12)	0.0195 (3)	
N4	0.24313 (19)	0.38636 (15)	0.73851 (12)	0.0207 (3)	
N5	−0.04970 (19)	0.77968 (15)	0.73930 (13)	0.0232 (3)	
C1	0.6620 (2)	0.57862 (16)	0.75956 (13)	0.0128 (3)	
C2	0.7799 (2)	0.47088 (16)	0.78859 (13)	0.0146 (3)	
C3	0.8485 (2)	0.39566 (17)	0.70360 (14)	0.0166 (3)	
H3	0.928	0.3205	0.7195	0.02*	
C4	0.7992 (2)	0.43239 (17)	0.59769 (14)	0.0182 (3)	
H4	0.845	0.383	0.5397	0.022*	
C5	0.6813 (2)	0.54268 (16)	0.57561 (14)	0.0158 (3)	
H5	0.6486	0.5672	0.5019	0.019*	
C6	0.8253 (2)	0.44452 (17)	0.90081 (14)	0.0171 (3)	
H6	0.905	0.3712	0.9219	0.021*	
C7	0.5862 (2)	0.65973 (16)	0.84136 (13)	0.0128 (3)	
C8	0.6326 (2)	0.63354 (16)	0.94977 (13)	0.0151 (3)	
C9	0.5529 (2)	0.71780 (17)	1.02540 (14)	0.0174 (3)	
H9	0.5812	0.705	1.0999	0.021*	
C10	0.4343 (2)	0.81839 (17)	0.99064 (14)	0.0180 (3)	
H10	0.3791	0.875	1.0412	0.022*	
C11	0.3953 (2)	0.83697 (16)	0.87987 (13)	0.0157 (3)	
H11	0.3125	0.9063	0.8567	0.019*	
C12	0.7560 (2)	0.52296 (17)	0.97732 (14)	0.0180 (3)	
H12	0.7895	0.5045	1.0506	0.022*	
C13	0.1266 (2)	1.13270 (17)	0.57670 (14)	0.0190 (3)	
H13A	0.0297	1.0988	0.632	0.028*	
H13B	0.0922	1.1839	0.5043	0.028*	
H13C	0.1812	1.1938	0.6083	0.028*	
C14	0.2445 (2)	1.01218 (16)	0.55433 (13)	0.0140 (3)	
C15	0.2838 (2)	0.98785 (16)	0.44328 (13)	0.0152 (3)	
H15	0.2332	1.0492	0.3847	0.018*	
C16	0.3917 (2)	0.88016 (16)	0.41157 (13)	0.0134 (3)	
C17	0.4305 (2)	0.86863 (17)	0.28774 (13)	0.0171 (3)	
H17A	0.5449	0.89	0.2564	0.026*	
H17B	0.3564	0.934	0.2433	0.026*	
H17C	0.4154	0.7744	0.2828	0.026*	
C18	0.2200 (2)	0.50592 (17)	0.71782 (13)	0.0152 (3)	
C19	0.0677 (2)	0.70993 (16)	0.71876 (14)	0.0163 (3)	
O1W	0.1223 (2)	0.12775 (15)	0.88013 (13)	0.0353 (4)	
O2W	−0.13527 (19)	0.96983 (16)	0.89006 (12)	0.0292 (3)	
H1A	0.165 (3)	0.195 (2)	0.8385 (17)	0.054 (8)*	
H1B	0.137 (3)	0.117 (2)	0.9432 (13)	0.034 (7)*	
H2A	−0.059 (3)	1.016 (2)	0.877 (2)	0.044 (8)*	
H2B	−0.116 (3)	0.9122 (19)	0.8518 (18)	0.041 (7)*	

### Atomic displacement parameters (Å^2^)

**Table:**

	*U*^11^	*U*^22^	*U*^33^	*U*^12^	*U*^13^	*U*^23^
Cu1	0.01465 (11)	0.01279 (10)	0.01157 (10)	0.00093 (7)	−0.00271 (7)	−0.00316 (7)
O1	0.0164 (6)	0.0138 (5)	0.0137 (5)	−0.0002 (5)	−0.0029 (4)	−0.0026 (4)
O2	0.0184 (6)	0.0135 (5)	0.0135 (5)	0.0005 (5)	−0.0035 (4)	−0.0033 (4)
N1	0.0146 (7)	0.0136 (6)	0.0112 (6)	−0.0029 (5)	−0.0013 (5)	−0.0026 (5)
N2	0.0155 (7)	0.0118 (6)	0.0129 (6)	−0.0011 (5)	−0.0010 (5)	−0.0026 (5)
N3	0.0163 (7)	0.0138 (7)	0.0267 (8)	−0.0019 (6)	0.0011 (6)	−0.0042 (6)
N4	0.0201 (8)	0.0182 (7)	0.0235 (7)	0.0007 (6)	−0.0052 (6)	−0.0037 (6)
N5	0.0179 (8)	0.0182 (7)	0.0317 (8)	−0.0021 (6)	−0.0017 (6)	−0.0033 (6)
C1	0.0118 (7)	0.0128 (7)	0.0141 (7)	−0.0028 (6)	−0.0015 (6)	−0.0031 (6)
C2	0.0130 (8)	0.0139 (7)	0.0167 (7)	−0.0023 (6)	−0.0028 (6)	−0.0021 (6)
C3	0.0142 (8)	0.0145 (8)	0.0216 (8)	0.0001 (6)	−0.0033 (6)	−0.0049 (6)
C4	0.0189 (9)	0.0173 (8)	0.0196 (8)	−0.0002 (7)	−0.0013 (6)	−0.0085 (6)
C5	0.0174 (8)	0.0167 (8)	0.0147 (7)	−0.0014 (6)	−0.0028 (6)	−0.0061 (6)
C6	0.0150 (8)	0.0179 (8)	0.0181 (8)	−0.0004 (6)	−0.0053 (6)	−0.0016 (6)
C7	0.0133 (8)	0.0130 (7)	0.0120 (7)	−0.0018 (6)	−0.0020 (6)	−0.0022 (6)
C8	0.0155 (8)	0.0165 (8)	0.0141 (7)	−0.0037 (6)	−0.0025 (6)	−0.0035 (6)
C9	0.0196 (9)	0.0214 (8)	0.0128 (7)	−0.0048 (7)	−0.0020 (6)	−0.0056 (6)
C10	0.0216 (9)	0.0189 (8)	0.0145 (7)	−0.0020 (7)	−0.0006 (6)	−0.0072 (6)
C11	0.0177 (8)	0.0137 (7)	0.0154 (7)	−0.0016 (6)	−0.0011 (6)	−0.0040 (6)
C12	0.0176 (9)	0.0212 (8)	0.0158 (8)	−0.0014 (7)	−0.0061 (6)	−0.0025 (6)
C13	0.0186 (9)	0.0177 (8)	0.0201 (8)	0.0034 (7)	−0.0031 (6)	−0.0054 (6)
C14	0.0119 (8)	0.0126 (7)	0.0175 (8)	−0.0029 (6)	−0.0017 (6)	−0.0026 (6)
C15	0.0159 (8)	0.0153 (7)	0.0143 (7)	0.0001 (6)	−0.0053 (6)	−0.0014 (6)
C16	0.0121 (8)	0.0151 (7)	0.0139 (7)	−0.0048 (6)	−0.0030 (6)	−0.0024 (6)
C17	0.0211 (9)	0.0178 (8)	0.0133 (7)	−0.0005 (7)	−0.0041 (6)	−0.0044 (6)
C18	0.0127 (8)	0.0223 (8)	0.0118 (7)	−0.0021 (6)	−0.0020 (6)	−0.0057 (6)
C19	0.0181 (8)	0.0142 (7)	0.0168 (8)	−0.0072 (6)	−0.0035 (6)	−0.0008 (6)
O1W	0.0484 (10)	0.0288 (8)	0.0272 (8)	−0.0153 (7)	−0.0032 (7)	0.0003 (7)
O2W	0.0272 (8)	0.0325 (8)	0.0322 (8)	0.0022 (7)	−0.0080 (6)	−0.0150 (6)

### Geometric parameters (Å, °)

**Table:**

Cu1—O1	1.9061 (11)	C6—H6	0.95
Cu1—O2	1.9072 (11)	C7—C8	1.394 (2)
Cu1—N1	2.0100 (14)	C8—C9	1.412 (2)
Cu1—N2	2.0136 (13)	C8—C12	1.440 (2)
Cu1—N3	2.3920 (15)	C9—C10	1.373 (2)
O1—C14	1.2775 (19)	C9—H9	0.95
O2—C16	1.2805 (19)	C10—C11	1.404 (2)
N1—C5	1.332 (2)	C10—H10	0.95
N1—C1	1.362 (2)	C11—H11	0.95
N2—C11	1.329 (2)	C12—H12	0.95
N2—C7	1.361 (2)	C13—C14	1.505 (2)
N3—C18	1.313 (2)	C13—H13A	0.98
N3—C19	1.324 (2)	C13—H13B	0.98
N4—C18	1.162 (2)	C13—H13C	0.98
N5—C19	1.154 (2)	C14—C15	1.395 (2)
C1—C2	1.396 (2)	C15—C16	1.398 (2)
C1—C7	1.436 (2)	C15—H15	0.95
C2—C3	1.413 (2)	C16—C17	1.503 (2)
C2—C6	1.435 (2)	C17—H17A	0.98
C3—C4	1.374 (2)	C17—H17B	0.98
C3—H3	0.95	C17—H17C	0.98
C4—C5	1.398 (2)	O1W—H1A	0.812 (15)
C4—H4	0.95	O1W—H1B	0.778 (15)
C5—H5	0.95	O2W—H2A	0.795 (15)
C6—C12	1.358 (2)	O2W—H2B	0.806 (15)
			
O1—Cu1—O2	95.58 (5)	C8—C7—C1	120.19 (14)
O1—Cu1—N1	171.80 (5)	C7—C8—C9	116.93 (15)
O2—Cu1—N1	90.01 (5)	C7—C8—C12	118.38 (15)
O1—Cu1—N2	91.52 (5)	C9—C8—C12	124.68 (15)
O2—Cu1—N2	168.73 (5)	C10—C9—C8	119.60 (15)
N1—Cu1—N2	82.08 (5)	C10—C9—H9	120.2
O1—Cu1—N3	89.74 (5)	C8—C9—H9	120.2
O2—Cu1—N3	94.16 (5)	C9—C10—C11	119.45 (15)
N1—Cu1—N3	95.85 (5)	C9—C10—H10	120.3
N2—Cu1—N3	94.62 (5)	C11—C10—H10	120.3
C14—O1—Cu1	124.52 (11)	N2—C11—C10	122.19 (15)
C16—O2—Cu1	124.19 (11)	N2—C11—H11	118.9
C5—N1—C1	118.31 (14)	C10—C11—H11	118.9
C5—N1—Cu1	128.97 (11)	C6—C12—C8	121.43 (15)
C1—N1—Cu1	112.72 (10)	C6—C12—H12	119.3
C11—N2—C7	118.32 (13)	C8—C12—H12	119.3
C11—N2—Cu1	129.06 (11)	C14—C13—H13A	109.5
C7—N2—Cu1	112.58 (10)	C14—C13—H13B	109.5
C18—N3—C19	119.10 (15)	H13A—C13—H13B	109.5
C18—N3—Cu1	123.48 (12)	C14—C13—H13C	109.5
C19—N3—Cu1	114.92 (11)	H13A—C13—H13C	109.5
N1—C1—C2	123.32 (15)	H13B—C13—H13C	109.5
N1—C1—C7	116.23 (14)	O1—C14—C15	125.18 (15)
C2—C1—C7	120.45 (14)	O1—C14—C13	115.20 (14)
C1—C2—C3	117.10 (15)	C15—C14—C13	119.63 (14)
C1—C2—C6	118.67 (15)	C14—C15—C16	125.02 (14)
C3—C2—C6	124.22 (15)	C14—C15—H15	117.5
C4—C3—C2	119.32 (15)	C16—C15—H15	117.5
C4—C3—H3	120.3	O2—C16—C15	125.37 (14)
C2—C3—H3	120.3	O2—C16—C17	114.54 (14)
C3—C4—C5	119.77 (15)	C15—C16—C17	120.08 (14)
C3—C4—H4	120.1	C16—C17—H17A	109.5
C5—C4—H4	120.1	C16—C17—H17B	109.5
N1—C5—C4	122.17 (15)	H17A—C17—H17B	109.5
N1—C5—H5	118.9	C16—C17—H17C	109.5
C4—C5—H5	118.9	H17A—C17—H17C	109.5
C12—C6—C2	120.86 (15)	H17B—C17—H17C	109.5
C12—C6—H6	119.6	N4—C18—N3	174.12 (19)
C2—C6—H6	119.6	N5—C19—N3	174.19 (18)
N2—C7—C8	123.49 (14)	H1A—O1W—H1B	112 (2)
N2—C7—C1	116.31 (13)	H2A—O2W—H2B	109.1 (19)

### Hydrogen-bond geometry (Å, °)

**Table:**

*D*—H···*A*	*D*—H	H···*A*	*D*···*A*	*D*—H···*A*
O2W—H2B···N5	0.81 (2)	2.08 (2)	2.879 (2)	173 (2)
O2W—H2A···O1W^i^	0.80 (2)	1.98 (2)	2.761 (2)	167 (2)
O1W—H1A···N4	0.81 (2)	2.10 (2)	2.910 (2)	172 (3)
O1W—H1B···O2W^ii^	0.78 (2)	2.00 (2)	2.742 (2)	160 (2)

Symmetry codes: (i) *x*, *y*+1, *z*; (ii) −*x*, −*y*+1, −*z*+2.
